# Circulating T Cell Subsets in Type 1 Diabetes

**DOI:** 10.3390/cells14010048

**Published:** 2025-01-04

**Authors:** Aldo Ferreira-Hermosillo, Paola Santana-Sánchez, Ricardo Vaquero-García, Manuel R. García-Sáenz, Angélica Castro-Ríos, Adriana K. Chávez-Rueda, Rita A. Gómez-Díaz, Luis Chávez-Sánchez, María V. Legorreta-Haquet

**Affiliations:** 1 Unidad de Investigación en Enfermedades Endócrinas de la UMAE Hospital de Especialidades, Centro Médico Nacional Siglo XXI, Instituto Mexicano del Seguro Social, Mexico City 06720, Mexico; aferreira@endocrinologia.org.mx; 2 Unidad de Investigación Médica en Inmunología, de la UMAE Hospital de Pediatría, Centro Médico Nacional Siglo XXI, Instituto Mexicano del Seguro Social, Mexico City 06720, Mexico; pao-ss@live.com.mx (P.S.-S.); ricardo-michelle@hotmail.com (R.V.-G.); akarina_chavez@yahoo.com.mx (A.K.C.-R.); luis_chz@hotmail.com (L.C.-S.); 3 Servicio de Endocrinología, de la UMAE Hospital de Especialidades, Centro Médico Nacional Siglo XXI, Instituto Mexicano del Seguro Social, Mexico City 06720, Mexico; manuel.gsm@hotmail.com; 4 Unidad de Investigación en Epidemiología Clínica de la UMAE Hospital de Pediatría, Centro Médico Nacional Siglo XXI, Instituto Mexicano del Seguro Social, Mexico City 06720, Mexico; angelica.castror@imss.gob.mx; 5 Unidad de Investigación en Epidemiología Clínica de la UMAE Hospital de Especialidades, Centro Médico Nacional Siglo XXI, Instituto Mexicano del Seguro Social, Mexico City 06720, Mexico; ritagomezdiaz@yahoo.com.mx

**Keywords:** type 1 diabetes, PD1, PRL, T cells

## Abstract

Type 1 diabetes (T1D) is a complex disease driven by the immune system attacking the insulin-producing beta cells in the pancreas. Understanding the role of different T cell subpopulations in the development and progression of T1D is crucial. By employing flow cytometry to compare the characteristics of T cells, we can pinpoint potential indicators of treatment response or therapeutic inefficacy. Our study reveals elevated prolactin (PRL) levels in T1D patients, along with a decreased production of key cytokines. Additionally, PD1 appears to play a significant role in T1D. Notably, PRL levels correlate with an earlier disease onset and a specific T cell phenotype, hinting at the potential influence of PRL. These findings highlight the need for further research to identify promising cellular targets for more effective and tailored therapies.

## 1. Introduction

Immune system activation is essential for pathogen control and disease prevention, but regulatory mechanisms are required to avoid immunopathology caused by overactive immune responses. Immune tolerance is a crucial mechanism that prevents immune responses against the body’s own antigens. However, an increase in the development of autoimmune diseases has been observed in recent years. These diseases are caused by harmful inflammatory responses involving innate immunity and autoreactive pathogenic T and B cells. T cells play a central role in regulating and initiating these responses. Therefore, understanding the mechanisms involved in autoimmune diseases and the therapeutic strategies being developed to restore immune tolerance is essential for determining the causes of autoimmune diseases such as type 1 diabetes (T1D).

According to the 2022 International Diabetes Federation report, 62% of newly diagnosed cases of T1D are individuals over the age of 20 [1]. This indicates that the development of T1D is significantly influenced by a complex interaction of environmental factors [2,3] involving the microbiome, genome, metabolism, and immune system. T1D is caused by the autoimmune destruction of pancreatic β-cells, leading to almost complete insulin deficiency resulting in hyperglycemia, and is associated with the inflammation and infiltration of lymphocytes [4,5].

At present, the standard treatment for T1D is insulin; however, it is not effective for many people in terms of optimally controlling glucose levels. Research in the field of T1D has led to the exploration of various therapies, including gene therapy, stem cells, and immunotherapy, which offer promise for personalized treatment. Recent clinical trials have focused on immune agents, such as the anti-CD3 antibody teplizumab, as well as agents that are believed to impact beta cells directly, such as verapamil. However, none of these agents have resulted in long-term disease remission [6]. As a result, new therapeutic approaches are being actively sought, even though they have not had the expected safety. For example, the use of PD-1/PD-L1-blocking antibodies has been approved for the treatment of more than 15 different types of cancer. However, it is important to note that up to 37% of patients treated with these antibodies develop immune-related adverse events, including T1D [7].

Programmed cell death protein-1 (PD-1; CD279) is a 55 kDa protein belonging to the immunoglobulin superfamily. It is a critical regulator of the adaptive immune system [8], and is expressed on the surface of activated T and B cells [7] upon chronic exposure to antigens in various infectious processes, autoimmune diseases, and cancer [9]. The interaction of PD-1 with its ligand, PDL-1, has an immunosuppressive effect on T cells through decreasing TCR signal transduction via its ITIM and ITAM motifs [10]. Studies have shown that a deficiency of PD-1 or PD-L1 results in profoundly accelerated autoimmunity [11]. Furthermore, PD-1 single nucleotide polymorphisms (SNPs) have been shown to increase the risk of developing T1D in several populations [12,13,14], suggesting that, at least in a subset of patients, PD-1 plays a key role in maintaining islet tolerance. Therefore, assessing the expression of this molecule in the different subpopulations of peripheral blood T cells could constitute a fundamental tool for predicting and monitoring T1D.

Although PD-1 is characterized as an inhibitory marker, several effector T cell populations are characterized by high expression of this marker. Among the various T cell subpopulations, PD1 is a marker generally associated with follicular T cells (Tfh) [15,16], regulatory T cells (Tregs), and exhausted T cells (Tex) [17], which are implicated in the progression of autoimmune diseases and possibly associated with the development of TD1. Follicular T cells represent the CD4+ T cell subset capable of supporting antibody production by B cells and the induction of immunoglobulin isotype switching [18], circulating Tfh cells (cTfh), involves memory CD4+ T cells that express CXCR5, but have low or absent BCL6 [19]. These cells are associated with a wide range of autoimmune diseases. Although there is no clear association with autoantibody production in T1D, Tfh cells from diabetic mice transferred the disease in a mouse model, and a Tfh cell signature has been identified in autoimmune diabetes, suggesting that this population could be used as a biomarker and potentially targeted for T1D interventions [20]; meanwhile, the increases in insulin-specific antigen cTfh cells [21] and activated cTfh cells (PD1hi) were significant in children with new-onset T1D or at risk for T1D [22].

On the other hand, chronic inflammation and high antigenic persistence identified in autoimmune diseases can lead to a state of cellular exhaustion. Exhausted T cells lose their normal functions, such as producing cytokines, killing cells, and multiplying, and start expressing multiple co-inhibitory receptors (CTLA-4, PD-1, LAG-3, TIM3, and TIGIT). This exhaustion happens when T cells are constantly exposed to an antigen. Recent findings have suggested that exhausted T cells might be linked to a better prognosis in T1D [23]. In addition, there are two relevant subtypes of T cells in T1D: central memory (CM; CD45RO+CCR7+) and effector memory (EM; CD45RO+CCR7-) T cells. Memory T cells can hold onto their protective function against familiar threats for many years without needing to be reminded of those threats [24]. Some studies have also found that memory T cells, particularly CD8+ T cells, have anti-tumor capabilities [25]. Also, memory T cells are thought to be one of the main contributors to the development of autoimmune diseases.

Moreover, it is well known that hormones such as prolactin (PRL) influence cellular responses. PRL is a hormone that stimulates cell growth and insulin production and may also enhance the development of blood vessels in pancreatic islets [26]. Additionally, PRL can function as a cytokine, influencing immune responses in various immune cell populations. However, its role in regulating the immune system is still controversial [27].

Research aiming to find effective treatments for T1D has shown that PRL treatment can enhance the engraftment and function of transplanted pancreatic islets, and it may prevent diabetes in patients treated with streptozotocin [28]. Nevertheless, the exact role of PRL in T1D remains unclear, and further studies are necessary for clarification, thus contributing to the discovery of new therapeutic strategies for the prognosis and monitoring of the development of T1D.

The main goal of this study is to analyze the different profiles of T cell subtypes and their potential association with serum PRL levels. We describe the frequency of exhausted T cells, Tfh cells, and memory T cells in patients with T1D to understand how hormonal factors and dysfunctional cellular responses could accelerate the development of autoimmunity.

## 2. Materials and Methods

A cross-sectional case-control study was developed to identify T cell subtype profiles associated with TD1. The cases group included patients with a diagnosis of T1D according to the criteria of the American Diabetes Association (ADA): those with clinical symptoms (polyuria, polydipsia and loss of unexplained weight, and ketoacidosis), biochemical data (fasting blood glucose ≥ 126 mg/dL, confirmed on a different day or blood glucose at any time of the day ≥200 mg/dL without considering the time of the last meal), requires the application of insulin for its treatment and had one or more markers of autoimmunity against the beta cell (e.g., antiGAD65, anti-IA2/-IA2ß or anti-Znt8), and without a history of neoplastic, chronic inflammatory disease or active infectious process. The control group included healthy 25- to 44-year-old volunteers without diabetes and without a history of a neoplasm, chronic inflammatory disease, or active infectious process.

### 2.1. Control Group

Healthy 25 to 44-year-old volunteers were selected, without diabetes and without a history of a neoplasm, chronic inflammatory disease, or active infectious process.

### 2.2. Sample Selection

A sample of 28 cases and 29 controls were randomly selected from patients treated in the T1D Clinic of the Endocrinology Service at the Hospital de Especialidades, Centro Médico Nacional Siglo XXI, a tertiary referral center. Group size estimates were based upon a power calculation to minimally yield an 80% chance to detect a significant difference in the respective parameter of *p* ≤ 0.05 between the relevant groups.

### 2.3. Reagents and Antibodies

A human prolactin ELISA kit (Fine Test, Wuhan, China) was used.

### 2.4. Blood Samples

Blood samples were collected from patients diagnosed with T1D and from healthy individuals. The samples were collected in tubes with K2 EDTA and without anticoagulant. From the anticoagulated blood, peripheral blood mononuclear cells (PBMCs) were separated using Lymphoprep (Axis-Shield, Liverpool, UK). The PBMCs were then recovered from the interface and washed three times with PBS (pH 7.4). Plasma was separated by centrifugation and kept for the determination of plasma PRL levels. The serum was also separated by centrifugation and stored at −20 °C for the determination of cytokines.

### 2.5. Biochemical Determinations

Laboratory results were obtained from the Central Laboratory at UMAE Hospital de Especialidades, C.M.N. Siglo XXI, IMSS. Briefly, a 6 mL blood sample was centrifuged at 3150× *g* for 15 min, and the serum was divided into two aliquots. Glucose, cholesterol, c-HDL, and triglycerides were analyzed with a commercially available kit (COBAS 2010 Roche Diagnostics, Indianapolis, IN, USA) using photocolorimetry with a Roche Modular P800 spectrophotometer (2010 Roche Diagnostics, Indianapolis, IN, USA). c-HDL samples were treated with enzymes modified with polyethylene glycol and dextran sulfate and analyzed using the same photocolorimetric technique. Glycated hemoglobin (HbA1c) was evaluated through turbidimetric immunoanalysis (COBAS 2010 Roche Diagnostics, Indianapolis, IN, USA). Low-density lipoprotein cholesterol (c-LDL) was calculated with the Friedewald formula, c-LDL (mg/dL) = CT mg/dL − (c-HDL mg/dL + triglycerides mg/dL/5), if triglycerides were <400 mg/dL.

### 2.6. Plasma PRL Levels

Prolactin in plasma was measured using a human PRL (Prolactin) ELISA kit (FineTest, Wuhan, China).

### 2.7. Serum Cytokine Levels

The levels of the cytokines IL-2, IFNγ, IL-4, IL-17, IL-10, IL-6, TNF-α, sFAS, sFAS-L, Granzymes A and B, perforin, and granulysin were determined in previously frozen sera using the Multi-analyte Flow Assay Kit (Biolegend, San Diego, CA, USA), according to the manufacturer’s instructions. To determine serum concentrations, the samples were analyzed on an MACS Quant X cytometer (Myltenyi Biotech, Santa Barbara, CA, USA).

### 2.8. Spectral Flow Cytometry

PBMCs were isolated from T1D patients and healthy donors via density centrifugation using Lymphoprep (Axis-Shield, Liverpool, UK). The PBMCs were recovered from the interface and washed three times with PBS (pH 7.4). The PBMCs were then stained with specific combinations of backbone antibody panels from the T cell subtype (Anti-human CD3-BV650 (OKT3), CD4-PE/F810 (SK3), CD8-APC/F810 (SK1), CD45RO-BV570 (UCHL1), CCR7-BV711 (G043H7), TIGIT-BV480 (A5153G), CTLA-4-PE (BNI3), LAG-3-BV785 (11C3C65), CXCR5-PE/F700 (J252D4), PD1SpkR718 (A17188B), and ICOS-PE (C398.4A), (Biolegend, San Diego, CA, USA)), for 20 min at 4 °C in the dark, and Ghost Dye, an amine reactive viability dye (Cytek^®^ Biosciences, San Diego, CA, USA), according to the manufacturer’s instructions. The cells were then washed three times with PBS, fixed with 2% paraformaldehyde, and analyzed using a Cytek^®^ Aurora system cytometer (Cytek^®^ Biosciences, San Diego, CA, USA).

### 2.9. Statistical Analysis

A descriptive and comparative analysis of the clinical characteristics of the groups was performed. Results are presented as mean (SD) or as percentages, where appropriate, for normally distributed data, and Student’s *t*-test for unpaired values was used to compare means between independent groups and the non-parametric Wilcoxon signed-ranks test, and the Mann–Whitney U tests were used to compare medians, mainly due to the sample size. A *p*-value of ≤0.05 was considered significant with a 95% CI.

To assess the relationship between T1D evolution time and cellularity, we conducted a Pearson correlation analysis to examine linear relationships. Additionally, we performed a nonparametric Spearman correlation test using a Bonferroni-adjusted significance level. We presented a two-way dispersion graph along with its polytomous adjustment. When necessary, we conducted a stratified analysis based on prolactin levels, categorizing them into normoprolactinemic and hyperprolactinemic groups. Prolactin levels were evaluated during the study and classified as normoprolactinemic (serum PRL levels < 20 ng/mL) or hyperprolactinemic (serum PRL levels > 20 ng/mL). Evolutionary time was defined as the duration between the sample collection date and the date of T1D diagnosis. For the control group, diagnosis time zero was designated.

Analyses were performed using GraphPad Prism 10 (La Jolla, CA, USA) and the Stata software (Version 11.0; Stata Corp., College Station, TX, USA).

### 2.10. Ethical Considerations

The study was approved by the Human Ethics and Medical Research Committee of the Mexican Institute of Social Security (IMSS) with register R-2018-785-074 and was conducted according to the guidelines of the Declaration of Helsinki. Informed consent was obtained from all patients and healthy donors.

## 3. Results

### 3.1. Demographic Characteristics of the Study Population

In this cross-sectional study, adult patients (age ≥ 18 years) diagnosed with T1D and age-matched healthy controls were included. All participants gave their consent to participate and donated a blood sample (10 mL). A total of 28 patients with T1D and 29 healthy controls were included. Of the total participants, 56% were female and 43% were male, with an average age of 30 years.

**Figure 1:**
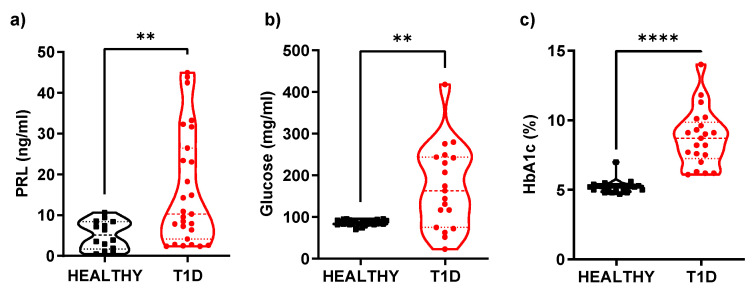
Serum levels of prolactin, glucose, and glycosylated hemoglobin. Quantification of serum levels of PRL glucose and HbA1 were compared between the patients with T1D and healthy controls. The results indicated significantly increased expression of (**a**) PRL (*p* = 0.007), (**b**) Glucose (*p* < 0.014), and (**c**) HbA1c (*p* < 0.0001) in T1D patients compared to healthy controls. Data are presented as mean ± standard division (SD). (Healthy controls, n = 29, PE women, n = 28). *p* < 0.05 is considered statistically significant (** *p* ≤ 0.01, **** *p* ≤ 0.0001).

As part of the characterization of the groups, serum levels of prolactin, HbA1c%, and glucose were measured and compared, with statistically significant differences (*p* ≤ 0.05) observed between the patient and control groups. Because it is a group with T1D, their glucose and HbA1c levels are higher than the control group: glucose (87 ± 6.4 mg/dL vs. 172 ± 100 mg/dL) and HbA1c (5.2 ± 0.43% vs. 8.7 ± 2.0%). Likewise, increased serum PRL levels were observed in the patients compared to healthy controls (16.2 ± 13.90 ng/mL vs. 5.19 ± 3.47 ng/mL; Table 1, Figure 1a). It should be noted that, although our group of patients was mostly comprised of women when performing the differential analysis of serum PRL concentrations between men and women, we did not find that gender influenced an increase in serum PRL concentration (T1D male 16.08 ± 13.3 ng/mL vs. T1D female 17.75 ± 14.70 ng/mL and healthy male 4.75 ± 2.42 ng/mL vs. healthy female 4.92 ± 3.65 ng/mL). A Spearman correlation analysis showed that there was a weak positive correlation between serum prolactin levels and %HbA1c (r = 0.3411, *p* = 0.038). This may indicate the relevance of the hormonal role, particularly of PRL, in the development and progression of the disease. The difference in PRL level expression between healthy donors and T1D patients could be a protective response to the pathology’s inherent damage or an alarming signal. An increase in serum PRL levels could be related to damage generation.

We found no differences regarding body mass index or waist circumference between the groups.

As we noticed an increase in serum levels of PRL, we decided to assess the hormonal profile of the study population. Although the role of hormones in autoimmunity is well-documented, our results did not show significant differences in the serum concentrations of hormones, such as estradiol, follicle-stimulating hormone (FSH), and free T4, contrary to our expectations. However, we observed a statistically significant increase in TSH (*p* = 0.031) in the group of patients with T1D compared to the control group. We also found higher testosterone levels in the control group, but all hormone levels were within normal ranges [27] (see Table 2). Table 2 provides mean, standard deviation, median, and interquartile range values for both study groups.

#### 3.1.1. Serological Features of the Study Population

When comparing the biochemical profiles, we did not find any significant differences between the two groups. From Table 3, it can be observed that the serum uric acid concentration was statistically significantly higher in the control group (6.18 ± 1.89 vs. 4.10 ± 1.10 mg/dL). This difference could be due to a less careful diet in the control group. However, the mean for both groups fell within the normal limits reported by the American Board of Internal Medicine: ABIM Laboratory Test Reference Ranges—January 2024 [29].

To further characterize our study population, we also determined serum cytokine levels to establish the predominant pro- or anti-inflammatory patterns in each group. We used an assay based on pre-coated beads with antibodies and flow cytometry to determine serum levels of various cytokines, such as IL-2, IL-4, IL-10, IL-6, TNF-α, sFAS, sFAS-L, Granzymes A and B, IFNγ, IL-17, perforin, and granulysin.

**Figure 2:**
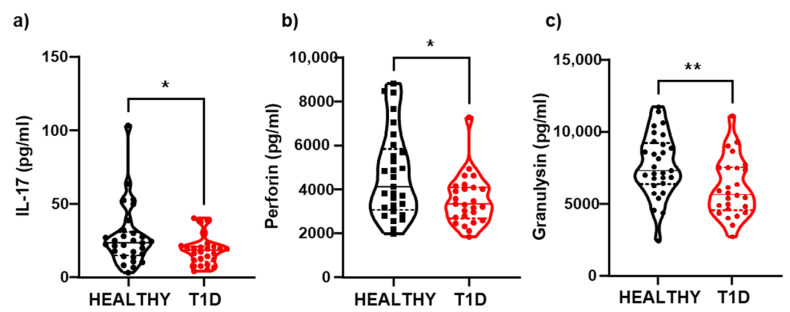
Serum levels of cytokines. Quantification of cytokine serum levels using flow cytometry. Serum cytokine levels were compared between patients with T1D and healthy controls. The results showed significantly increased expression of (**a**) IL-17 (*p* = 0.028), (**b**) Perforin (*p* < 0.028), and (**c**) Granulysin (*p* < 0.008) in healthy controls compared to T1D patients. Data is presented as mean ± standard deviation (SD). (Healthy controls, n = 29; T1D patients, n = 28). *p* < 0.05 was considered statistically significant (* *p* ≤ 0.05, ** *p* ≤ 0.01).

Our results showed that the healthy control group had elevated levels of certain cytokines, such as IL-17 (27.69 ± 20.5 vs. 18.05 ± 9.8 pg/mL), perforin (4651 ± 1970 vs. 3493 ± 1126 pg/mL), and granulysin (7668 ± 2177 vs. 6103 ± 2007 pg/mL), when compared to the group of patients with DM1 (Figure 2a–c). We did not observe significant differences for the rest of the cytokines determined, although, in all cases, the concentrations of cytokines tended to be higher in the control group. This could be due to a potential state of immune system exhaustion in patients with T1D due to antigenic persistence, given the chronic nature of the disease.

#### 3.1.2. Immunophenotype

**Figure 3:**
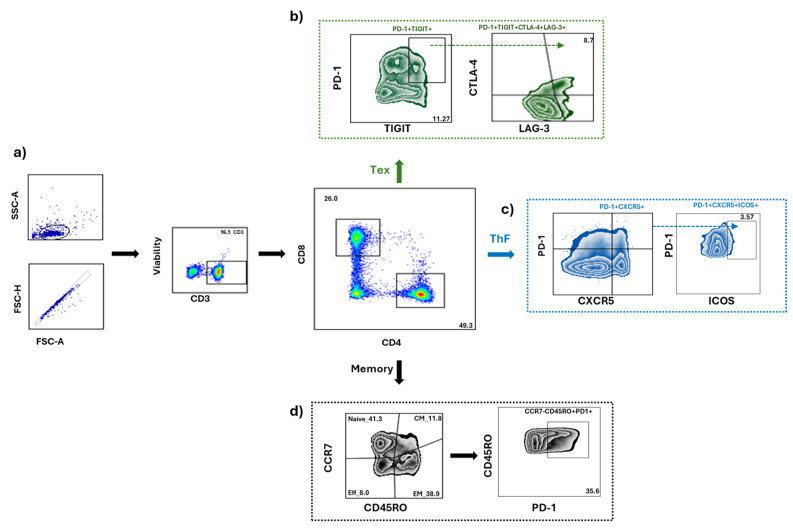
Circulating T cells. (**a**) Flow cytometry analysis strategy to gate on T cells. FSC-H vs. FSC-A plot was used to exclude doublets; lymphocytes were gated on the FSC-A vs. SSC-A plot, and live cells were gated in the Ghost Dye negative (Viability) and CD3-BV650+. From living T cells, we selected the CD4+ or CD8+ gate, and using the pre-designed panels from each T cell subtype, we selected the following markers: (**b**) Exhausted T cells (CD4+TIGIT+PD1+LAG3+CTLA-4+ or CD8+TIGIT+PD1+LAG3+CTLA-4+). (**c**) Follicular T cells (CD4+CXCR5+PD1+ICOS+ or CD8+CXCR5+PD1+ICOS+) (**d**) Memory T cells (CD4+CD45RO± CCR7± or CD8+CD45RO±CCR7±).

To understand the relevance of T cells, we decided to examine the immune status of our groups by analyzing the cellular phenotypes of circulating T cell subsets, particularly focusing on follicular T cells, exhausted T cells, and different memory T cell populations. We used spectral flow cytometry to determine the percentages and absolute numbers of these T cell subsets from peripheral blood mononuclear cells, following the analysis strategy shown in Figure 3.

**Figure 4:**
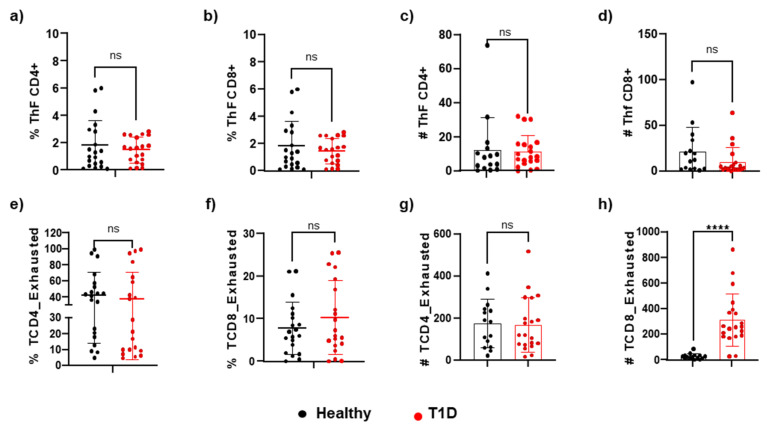
**Comparison of frequencies of CD4/CD8 among live CD3+.** (**a**–**d**) follicular T (CD4+CXCR5+PD1+ICOS+ or CD8+CXCR5+PD1+ICOS+), and (**e**–**h**) exhausted T (CD4+TIGIT+ PD1+LAG3+CTLA-4+ or CD8+TIGIT+PD1+ LAG3+CTLA-4+). These are shown as proportions or absolute numbers of circulating T cells in T1D and healthy controls. *p* < 0.05 was considered statistically significant (**** *p* ≤ 0.0001).

There were no significant differences in the percentages (Figure 4a,b) or absolute numbers (Figure 4c,d) of follicular T cells between the two groups. However, we observed a statistically significant increase in the number of exhausted CD8+ T cells in the group of patients with T1D compared to healthy controls (312.0 ± 205.7 vs. 27.73 ± 21.35 cells/µL) (Figure 4h).

**Figure 5:**
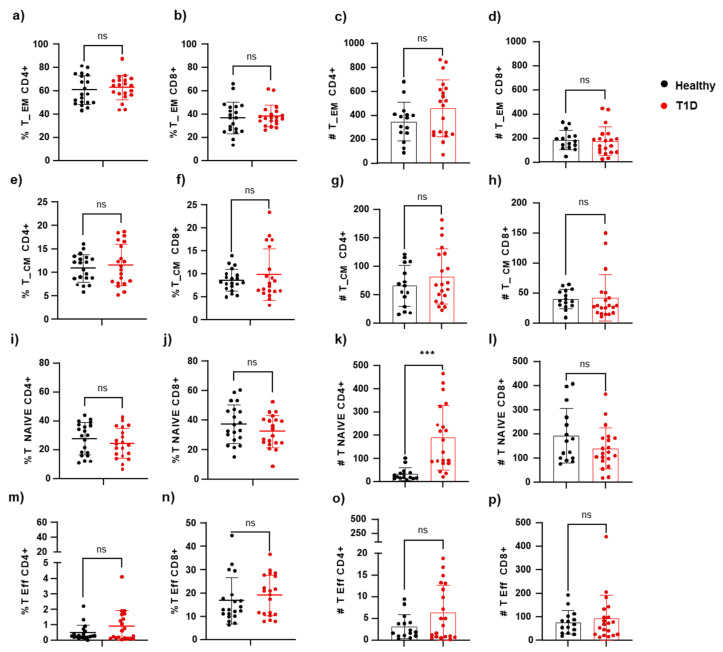
Comparison of the frequencies of CD4/CD8 among live CD3+ cells, TCD4+ or T CD8+. (**a**–**d**) effector memory (CD45RO+CCR7-), (**e**–**h**) central memory (CD4+CD45RO+CCR7+), (**i**–**l**) naive (CD45RO-CCR7+), and (**m**–**p**) effector (CD45RO-CCR7-) T cells are shown as proportions (left) or absolute numbers (right) of circulating T cells in T1D and healthy controls. *p* < 0.05 was considered statistically significant (*** *p* ≤ 0.001).

We examined if there were cellular changes in the memory cell fraction by calculating the percentage and number of CD4+ T cells or CD8+ T cells with specific phenotypes. We considered effector memory cells (CD45RO+CCR7-), central memory cells (CD4+CD45RO+CCR7+), effector cells (CD45RO-CCR7-), and naive cells (CD45RO-CCR7+). Our findings are presented in Figure 5a–p. Overall, we did not observe significant differences in the memory T cell compartment, except for an increase in the naive CD4+ T cell subpopulation in the T1D patient group compared to healthy individuals (82.18 ± 49.02 vs. 65.69 ± 36.32 cells/μL). We noted that peripheral naive T cells can be largely sustained by homeostatic expansion and tonic T cell receptor (TCR) signaling.

As there were no noteworthy differences in the T cell subtypes assessed, we sought to determine whether there was a functional difference in these cells beyond just numerical variations. We specifically focused on analyzing the expression levels of PD-1, an important immune checkpoint that negatively regulates the stability and integrity of T cell immune function, in the CD4+ T or CD8+ T cell subpopulations that we previously examined (Thf, memory T, and exhausted T).

**Figure 6:**
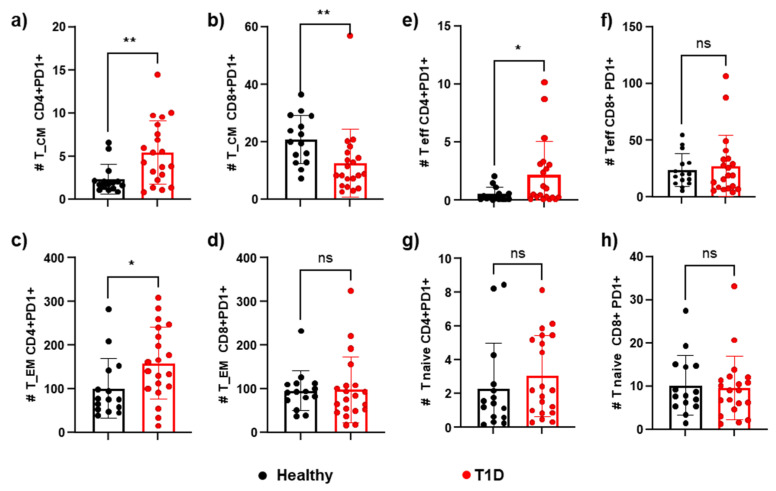
**Comparison of frequencies of TCD4+ or T CD8+PD1+ between those of** (**a**,**b**) central memory (CD4+CD45RO+CCR7+), (**c**,**d**) effector memory (CD4+CD45RO+CCR7-), (**e**,**f**) effectors (CD45RO-CCR7-), and (**g**,**h**) naïve (CD45RO-CCR7+) are shown as the absolute number of circulating T cells in T1D and healthy controls. *p* < 0.05 was considered statistically significant (* *p* ≤ 0.05, ** *p* ≤ 0.01).

As seen in Figure 6, there was a difference in the expression of PD1 between the memory T cell subpopulations. There was a statistically significant increase in PD1 expression in the T1D patient group, compared to the control group, in central memory CD4+ T cells (5.45 ± 3.67 vs. 2.35 ± 1.68 cells/μL), effector memory (159.4 ± 82.63 vs. 101.8 ± 68.54 cells/μL), and effector (2.19 ± 2.89 vs. 0.5141 ± 0.6044 cells/μL). This might indicate an attempt to restore balance in a system that is overactivated by autoimmune antigen persistence (Figure 6a,c,e). On the other hand, in CD8+ T cells, there was a statistically significant decrease in the central memory cell compartment in patients with T1D compared to the group of healthy individuals (20.87 ± 8.414 vs. 12.68 ± 11.88 cells/μL) (Figure 6b).

To analyze the relationships between cell populations and serum prolactin concentrations during the progression of T1D, we performed a Pearson correlation analysis (for linear relationships) and a Spearman rank analysis (for non-linear relationships). These analyses were stratified based on the level of prolactin control. The significance level of the correlation was assessed using the Bonferroni-adjusted statistical test.

Prolactin was evaluated at the time of the blood study and categorized as normoprolactinemia (serum PRL levels < 20 ng/mL) or hyperprolactinemia (serum PRL levels > 20 ng/mL). The evolution time was calculated as the duration between the date of sample collection and the date of T1D diagnosis confirmation. For the control group of individuals without diabetes, a diagnosis time of zero was specified.

**Figure 7:**
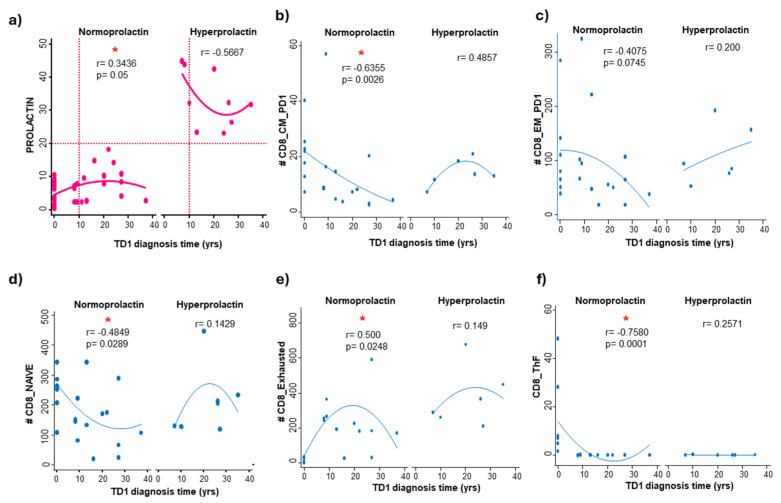
**Two-way dispersion graph: prolactin and CD4 vs. TD1 diagnosis time** (**a**) Spearman’s correlations_ TD1 diagnosis time and prolactin levels: NP r = 0.3436, *p* = 0.050 *, HP r = −0.5667, ns. (**b**) TD1 diagnosis time and CD8-CM: NP r = −0.6355, *p* = 0.0026 *, HP r = 0.4857, ns. (**c**) TD1 diagnosis time and CD8-EM_PD1: NP r = −0.4075, ns HP r = 0.200, ns (**d**) TD1 diagnosis time and CD8_NAIVE: NP r = −0.4849, *p* = 0.0289 * HP r = 0.1429, ns. (**e**) TD1 diagnosis time and CD8_Exhausted: NP r = 0.500, *p* = 0.024 *. HP r = 0.200, ns. (**f**) TD1 diagnosis time and CD8_ThF = −0.7580, *p* = 0.0001 *. HP r = 0.2571, ns. HP = Hyperprolactin, NP = Normoprolactin.

In Figure 7, the scatter plots illustrate the relationship between serum PRL levels and diagnosis time of T1D. In the hyperprolactinemic group, there was a strong negative correlation (r = −0.5667) between PRL levels and time since diagnosis, although it was not statistically significant. This suggests a potential involvement of prolactin in the etiopathogenesis of T1D. Conversely, in normoprolactinemic patients, there was a weak positive correlation (r = 0.3436, *p* = 0.050 *) between serum PRL levels and the time of diagnosis.

The analysis of the correlation between serum prolactin levels and time was also performed for all T cell subpopulations, revealing a strong negative correlation for CD8_CM_PD1 (r = −0.6355, *p* = 0.0026 *) (Figure 7b), a weak negative correlation for CD8_NAIVE (r = −0.4849, *p* = 0.0289) (Figure 7d), a strong positive correlation for CD8_Exhausted (r = 0.500, *p* = 0.024 *) (Figure 7e), and a strong negative correlation for CD8_Thf (r = −0.7580, *p* = 0.0001 *) (Figure 7f), in the normoprolactinemic group.

Significant correlations were not found in the hyperprolactinemic group. However, this group tends to increase cellular levels concerning the diagnosis time. This difference may warrant further investigation in the search for distinguishing markers that can help track the progression of the disease.

It is important to note that correlation does not imply causality, but we believe that this approach could help identify prognostic or monitoring markers in these patients.

Due to the complexity of the autoimmunity phenomenon and the variation in cellular profiles among patients with T1D, further study of these cellular subpopulations is necessary to confirm the identified profiles and evaluate the results associated with each patient group.

## 4. Discussion

The measurement of HbA1c is an important diagnostic tool for diseases, serving as the primary indicator of glycemic control. It correlates with the clinical picture and can predict the development of complications [30]. Due to the T1D, the patient group had significantly higher levels of HbA1c and glucose compared to the control group; this may indicate inadequate glycemic control and a predisposition to clinical complications [31]. However, other biochemical parameters of the patient group did not indicate metabolic or renal alterations, and we were unable to establish associations between the presence of obesity or overweight and the evaluated biochemical and cellular parameters.

In terms of the hormonal profile, we found a direct relationship between serum levels of PRL and HbA1c. Although the majority of the patients with T1D were women, there was no difference in the profile of steroid and thyroid hormones between the study populations, even when data were grouped according to gender.

Prolactin, a hormone secreted in the anterior pituitary, is involved in various physiological processes such as lactation, metabolism, or immunoregulation [32]. Increased levels of prolactin have been reported in various autoimmune diseases and were observed in the group of patients in this study, suggesting potential effects on the activation and secretion of cytokines in immune cells [33]. However, at present, there is insufficient evidence for the participation of this hormone in the pathophysiology of T1D.

Given the pivotal role of T cells in the development of T1D, it is crucial to further investigate the potential alterations in the various T cell populations. However, this is a challenging task due to the inherent complexity of these cells. For instance, autoreactive T cells have specific characteristics that make it challenging to develop biomarkers for T cells in diabetes, such as their low frequency in peripheral blood and their minimal response to the peptide–MHC complex. Furthermore, healthy individuals carrying MHC molecules associated with a risk for T1D may have autoreactive T cells that are quantitatively and functionally similar to those of patients with T1D [34]. Additionally, during activation, CD4+ T cells undergo less efficient metabolic reprogramming in aerobic glycolysis, similar to highly proliferative cancer cells. To limit tumor growth in cancer, glycolytic inhibitors are used, and this strategy has also been employed to suppress T cell responses in autoimmune diseases such as systemic lupus erythematosus, multiple sclerosis, and rheumatoid arthritis. However, the modulation of T cell metabolism in the context of T1D remains an underexplored therapeutic opportunity [35]. Multiple mechanisms are involved in maintaining peripheral immune tolerance. Immune checkpoint inhibitors play a vital role in immune tolerance by downregulating the immune system. Among these, PD-1 and PD-L1 are essential for the termination of immune responses. The PD1/PDL1 pathway induces immune system tolerance by promoting the development of Tregs and suppressing effector T cell responses [36].

In this study, the frequency and number of different CD4+ and CD8+ T cell subpopulations were evaluated to search for prognostic markers and their associations with the broad spectrum of clinical manifestations of the disease. Knowledge of the phenotype and function of cells is a fundamental tool in the search for new therapeutic approaches. The obtained results revealed discrete changes in the phenotype of T cell subpopulations and some effector molecules such as PD1. PD1 leads a highly relevant pathway due to its inhibitory functions in chronic viral infections and tumors and has special relevance in the context of autoimmunity. It is known that the use of PD-1 antagonist monoclonal antibodies is a therapeutic alternative in various neoplasia that has presented favorable effects in different clinical phases [37]. However, cases of spontaneous development of T1D in patients have been reported, with the rapid development of ketoacidosis and formation of autoantibodies in almost half of the patients who presented T1D [38,39]. In fact, dual use of antibodies against CTLA-4 and PD-1 or PD-L1 has been associated with an increased risk of developing T1D (hazard ratio [HR] = 1.62), compared to the use of anti-PD-L1 or anti-PD-1 alone [38]. Therefore, the association of this molecule with pathology is relevant.

In patients with T1D, the population of exhausted CD8 T cells is increased compared to that in healthy individuals. This increase could be due to the persistence of antigens as a result of the chronic nature of the disease. This highlights exhausted T cells as an important profile to monitor the development or progression of T1D. CD8+ cells have the ability to release antimicrobial and cytolytic molecules, such as granulysin and perforin, during infectious and autoimmune processes, contributing to the maintenance of inflammation [40,41,42]. However, studies in mice have shown that perforin’s cytolytic activity leads to the destruction of β cells [43]. When comparing the serum levels of these molecules and pro- and anti-inflammatory cytokines in T1D patients and healthy individuals, our results indicated significantly lower levels of perforin and granulysin in the T1D patient group. This suggests that constant antigen presentation during the autoimmune process may cause an exhausted state, resulting in a decrease in effector response capacity, such as the secretion of granules and proinflammatory cytokines [44,45]. Furthermore, there is also a significant decrease in IL-17—a pro-inflammatory cytokine—in patients with T1D, which is consistent with the increase in exhausted CD8+ T cells.

Our findings indicated an increase in the expression of CD8+ TIGIT+ potentially exhausted, while cells CD4+ TIGIT+ did not show a similar increase. The rise in the frequency of CD8+ exhausted T cells has been linked to slower disease progression. Furthermore, it has been observed that patients with T1D who responded effectively to teplizumab (CD3) treatment had a higher percentage of partially exhausted CD8+ EOMES+ KLRG1+ TIGIT+ T cells [46].

Regarding serum PRL levels, in cases where prolactin levels are normal, serum PRL remains consistent regardless of the age at diagnosis. Additionally, patients with normal prolactin levels showed minor levels of naive T CD8+ cells, effector memory cells, central memory cells, and ThF cells, which seem to be linked to early ages at diagnosis.

It is known that hyperprolactinemia has been associated with the development of various autoimmune diseases, such as systemic lupus erythematosus, potentially affecting disease activity. Research from both clinical and experimental studies indicates that the impact of prolactin on metabolism and the immune response varies based on its levels in the blood. Some studies suggest that prolactin has a protective effect. In contrast, others indicate that it could act as a pro-inflammatory factor, leading to harmful metabolic and immune changes associated with hyperprolactinemia.

PRL’s role is still controversial. It has been shown in some human or rat experiments to stimulate glucose-dependent insulin secretion, insulin gene transcription, and β-cell proliferation [47,48]. Conversely, in some people, poor insulin secretion has been linked to chronic hyperprolactinemia [49,50]. Further research is required to better understand how excess or deficiency of PRL affects metabolic changes and to look into the possibility of using PRL as a therapeutic target.

Currently, there is insufficient information regarding the connection between PRL and T1D. Research, including our own, would facilitate a more in-depth exploration of the potential role of the hormone PRL in T1D. Regarding the correlation analyses, while we understand the necessity for a larger sample size and more extensive analysis, these methods could be adapted and employed in prediction or therapeutic monitoring models. Specifically, we could investigate the relationships between serum prolactin levels and CD8 T cell subtypes (e.g., naïve or follicular) in order to better monitor and understand the progression of T1D.

The study’s strengths include the generation of knowledge in a fertile field that is challenging due to the limited experimental information available in the literature. Thus, there is a need to propose new analytical approaches that could lead to innovative strategies for monitoring T1D. This could involve considering the dual character of PRL as a growth factor with immunomodulatory activity and exploring the combination of hormonal and immunological approaches. It is important to acknowledge that extensive research in this field. The use of spectral flow cytometry as a powerful tool for analyzing cellular populations offers the possibility of multivariate analysis, allowing us to construct marker panels that could potentially be useful as prognostic or therapeutic sufficiency markers. As for the weaknesses of the study, we can mention the limited number of patients and controls. However, as an initial approach, we obtained a general overview of the cellular populations ex vivo, which provides a basis for further study through functional analysis of these cellular subtypes in evaluating autoreactive responses. The reported phenotypic observations justify future specific studies on the impacts of the observed expression levels.

## 5. Conclusions

The presence of follicular T cells in both T1D patients and control subjects suggests that this cell type may not play a crucial role in the clinical course of T1D. However, differences in the frequencies of exhausted T cells indicate their potential connection to the development or progression of T1D, making them promising targets for therapeutic interventions. Our study demonstrated that the expression of immune checkpoints in the peripheral blood differs in patients with T1D when compared to healthy individuals, implying a potential suboptimal adaptation of the immune response. This underscores the importance of exploring the potential clinical applications of PD-1 manipulation in the context of autoimmunity. We cannot dismiss the possible impact of PRL on the response of memory T cells.

As a result, further immunological assessments of the phenotype and function of relevant T cell subtypes are essential for more accurate prediction, monitoring, and classification of patients with T1D. The ultimate goal is to explore how signaling pathways involving PD-1 could be harnessed to modulate the immune response and potentially prevent or treat autoimmune diseases such as T1D.

## Figures and Tables

**Table 1:** Demographic characteristics.

Characteristic	Healthy	T1D	Total	*p* Value
	(n = 29)	(n = 28)	(n = 57)	
Sex—no. (%)				0.047
Male	16 (55.2%)	9 (32.14%)	25 (43.85%)	
Female	13 (44.8%)	19 (67.86%)	32 (56.15%)	
Age group—no.	29.8 (25–44)	32.03 (18–43)	30.63 (18–44)	0.024
Serum PRL (ng/mL)	5.19 ± 3.47	16.2 ± 13.90		0.007 *
HbA1c %	5.2 ± 0.43	8.7 ± 2.0		<0.001 *
Serum Glucose (mg/mL)	87 ± 6.4	172 ± 100		0.014 *
Waist circumference	90.54 ± 17.65	83.61 ± 9.87		0.325
Body-mass index (kg/m^2^)	25.42 ± 5.21	25.21 ± 3.65		0.731
≥30.0: obese	7 (24.14%)	4 (14.28%)	11 (19.29%)	
Age onset (years)		15.0 ± 8.11		
Duration of diabetes (years)		17.0 ± 8.68		

Values represented as mean ± SD. *p* ≤ 0.05 was considered statistically significant (*). PRL: Prolactin. HbA1c %: Percentage of glycated hemoglobin.

**Table 2:** Hormonal profile.

Group	Control		T1DM		Control		T1DM		*p* *
Statistics	Mean	SD	Mean	SD	Median	IQR ^a^	p50	IQR ^a^	Control vs. T1DM
Estradiol (pg/mL)	82.0	92.3	77.5	52.1	42.6	69.6	60.0	72.9	0.794
Testosterone (ng/dL)	403.3	302.9	199.7	282.1	468.6	500.3	36.3	477.7	0.034
FSH (MU/mL)	5.2	3.7	5.5	4.2	3.9	4.4	4.4	3.2	0.513
LH (MU/mL)	6.9	9.6	6.8	4.9	4.8	3.4	4.7	7.8	0.751
TSH (MU/mL)	2.0	0.8	2.6	1.5	1.8	1.1	2.9	3.1	0.031
T4 (ng/mL)	1.3	0.2	1.9	2.8	1.3	0.2	1.3	0.3	0.641

Values represented as mean ± SD and median ± IQR (q75-q25). *p* ≤ 0.05 was considered statistically significant. FSH = Follicle-stimulating hormone, LH = Luteinizing Hormone. TSH = Thyroid stimulating hormone (thyrotropin), T4 = Thyroxine 4. * Two-sample Kolmogorov–Smirnov test for equality of distribution functions. ^a^: IQR = (q75-q25).

**Table 3:** Biochemical profile.

	Healthy	T1D	*p* Value
	(n = 22)	(n = 24)	
Cholesterol (mg/dL)	179.9 ± 34.59	166.6 ± 36.97	0.28
cHDL (mg/dL)	48.81 ± 9.125	48.75 ± 8.779	0.785
cLDL (mg/dL)	107.1 ± 28.24	93.66 ± 34.58	0.219
Triglycerides (mg/dL)	121.1 ± 81.13	111.6 ± 52.51	0.657
Uric acid (mg/dL)	6.181 ± 1.899	4.100 ± 1.107	<0.001
Creatinine (mg/dL)	81.99 ± 92.30	281.1 ± 847.1	0.621

Values represented as mean ± SD. *p* ≤ 0.05 was considered statistically significant. cHDL: High-density lipoprotein cholesterol, cLDL: Low-density lipoprotein cholesterol.

## Data Availability

The data presented in this study are available on request from the corresponding author.
